# Peach Peel Extrusion for the Development of Sustainable Gluten-Free Plant-Based Flours

**DOI:** 10.3390/molecules30030573

**Published:** 2025-01-27

**Authors:** Ana Belen Martín-Diana, Iván Jesús Jiménez-Pulido, Ingrid Aguiló-Aguayo, Maribel Abadías, Jara Pérez-Jiménez, Daniel Rico

**Affiliations:** 1 Agrarian Technological Institute of Castilla and Leon (ITACyL), Ctra. Burgos Km 119, Finca Zamadueñas, 47071 Valladolid, Spain; jimpuliv@itacyl.es; 2 IRTA, Postharvest, Fruitcentre, 25003 Lleida, Spain; ingrid.aguilo@irta.cat (I.A.-A.); isabel.abadias@irta.cat (M.A.); 3 Department of Metabolism and Nutrition, Institute of Food Science, Technology and Nutrition (ICTAN-CSIC), Jose Antonio Novais 10, 28040 Madrid, Spain; jara.perez@ictan.csic.es; 4 CIBER de Diabetes y Enfermedades Metabólicas Asociadas (CIBERDEM), Instituto de Salud Carlos III (ISCIII), 28029 Madrid, Spain; 5 Endocrinology and Clinical Nutrition Research Center (CIENC/IENVA), Faculty of Medicine, University of Valladolid, Av. Ramón y Cajal, 7, 47005 Valladolid, Spain

**Keywords:** peach, peel, flours, extrusion, gluten free, functionality, antioxidant, valorization

## Abstract

The food industry generates substantial waste, contributing to environmental challenges, such as pollution and greenhouse gas emissions. Utilizing by-products, particularly fruit peels that are rich in fiber, antioxidants, and vitamins, presents a sustainable approach to reducing waste, while enhancing the nutritional value of food products. Specifically, peach peel can be used to produce gluten-free flours, with increased fiber content and antioxidant properties. Extrusion technology is a highly effective method for developing these functional flours, as it improves digestibility, reduces anti-nutrients, and enhances nutrient bioavailability. This study investigates the potential of combining corn flour with peach peel flour, derived from Royal Summer peachs (RSF), at different concentrations (0%, 5%, and 15%). A factorial experimental design was utilized to evaluate the impact of RSF incorporation on the proximate composition, antioxidant capacity, and functional properties of the flour. The results indicate that flours containing 15% RSF demonstrated significant improvements in terms of the dietary fiber content (5.90 g per 100 g^−1^) and antioxidant capacity (ABTS^•+^ 745.33 µmol TE per 100 g^−1^), meeting the “source of fiber” labelling requirements. The glycemic index of the 15% RSF flour was reduced to 78.09 compared to non-enriched flours. The functional properties of the flour, such as swelling and gelation capacities, were also enhanced with RSF incorporation. These findings highlight the potential of RSF-enriched flours in regard to the development of sustainable, health-promoting, plant-based, and gluten-free flours.

## 1. Introduction

The agri-food sector generates a substantial amount of waste, presenting significant environmental, economic, and social challenges [1]. Inefficient waste management contributes to 19–29% of soil and water contamination and increases greenhouse gas emissions from organic matter decomposition [2,3]. To address these issues, the United Nations’ 2030 Agenda for Sustainable Development emphasizes the importance of responsible consumption and production, including the reduction of per capita food waste [4].

The valorization of by-products from the agri-food industry has emerged as a sustainable strategy to achieve these objectives, while simultaneously reducing raw material costs [2,5]. Fruit processing, in particular, generates considerable by-products, such as peel, seeds, and pulp, which remain underutilized, despite their high concentrations of bioactive compounds and nutrients. These by-products are rich in antioxidants (e.g., flavonoids, phenolic acids), dietary fiber, vitamins, and essential fatty acids, rendering them suitable for applications in functional foods, nutraceuticals, and animal feed [6,7,8,9,10,11,12,13,14].

This approach to converting waste into value-added products aligns with the growing consumer interest in plant-based and nutrient-dense diets, who are increasingly motivated by concerns regarding health, sustainability, and ethics [15]. This trend is especially pronounced among younger generations, driving innovation in plant-based food production [16,17,18].

Nectarine (*Prunus persica var. nucipersica*) and peache (*Prunus persica L. Batsch*) are among the most economically significant fruit crops globally, ranking after apples (*Malus spp*.) and pears (*Pyrus* spp.) [19]. These fruits are cultivated on over 1.5 million hectares of land worldwide, with a production volume of 26.35 million tons in 2022. The leading producers of these fruit crops include China, Italy, Turkey, Greece, and Spain [19]. While peachs are primarily consumed fresh, they are also processed into products such as jams, juices, dried fruits, and yogurt [20].

Fruit processing generates considerable waste, including skin (22.5% of the fruit’s weight) and kernels (5–12.5%), resulting in waste volumes equivalent to 27.5–35% of the total production volume. This corresponds to an estimated 7.25–9.23 million tons of waste annually, with part of this material being discarded or utilized for animal feed [21]. Peach peel, rich in dietary fiber, phenolic compounds, vitamins, and minerals, is particularly promising in regard to the development of fiber-enriched products aimed at improving digestive health and reducing the risk of chronic diseases, such as type 2 diabetes and cardiovascular conditions [22,23,24,25]. In Europe, where the average daily fiber intake is approximately 18 g, which is below the Food and Agriculture Organization’s (FAO) recommended 25–30 g/day, such flours could help address dietary fiber deficiencies [26,27,28].

The increasing prevalence of celiac disease and gluten sensitivity has further heightened the demand for gluten-free flours. Traditional gluten-free flours, such as rice and maize, often lack fiber and essential nutrients [29,30]. The incorporation of fruit by-products, coupled with advanced processing techniques, such as extrusion, offers a solution to enhance the nutritional profile of gluten-free flours [31,32,33].

Different studies have mentioned that the extrusion process is an interesting technology in regard to the development of new gluten-free flours [34,35,36,37]. Delgado-Murillo et al. [38] determined the physicochemical, nutritional, and cooking properties, after extrusion, of gluten-free pasta, made from a mixture of rice and chickpea flour, and concluded that the extrusion process enables the incorporation of different flours, such as chickpea flour, increasing the content of phenolic compounds and dietary fiber. Caporizzi et al. [39] studied different variables during the extrusion process involving a mixture of teff and rice flour, such as the percentage of teff flour, the feed moisture, and process temperature, and determined the techno-functional and nutritional properties of the flour, obtaining a higher fiber content and antioxidant capacity when increasing the percentage of teff flour.

The extrusion process is an important technology for the food industry that is used to modify the physical and functional properties of molecules, such as starch and proteins. The extrusion process combines the application of high temperature, pressure, and shear stress, to modify the molecular structure of food components, which affects their functional and nutritional properties [40]. In particular, extrusion causes starch gelatinization and protein denaturation, which improves the digestibility of these compounds.

In addition, the extrusion process reduces the amount of anti-nutrients present in foods, increasing the bioavailability of essential nutrients, such as minerals and vitamins [41,42]. Recent studies have shown the ability of extrusion to optimize the nutritional quality of flours [34,35,38,39].

One of the main effects of extrusion on starch is gelatinization and posterior dextrinization, causing the rupture of the crystalline structure and the release of amylose and amylopectin molecules. These structural changes significantly increase the solubility of starch and make it more susceptible to the action of digestive enzymes, resulting in the production of a source of energy [43]. Additionally, during the extrusion process, the formation of oligosaccharides and hydroxyl groups improves the ability of starch to interact with proteins [44,45]. This interaction increases the emulsifying capacity and stability of food products, which is especially useful in the manufacture of gluten-free flours and other functional ingredients [46,47]. Recent studies have shown that extrusion can modify the structure of starch in a controlled way to produce products with different functional characteristics, which opens up new possibilities in regard to the development of innovative foods [48,49].

Proteins undergo significant changes during extrusion, including denaturation and subunit aggregation, which impact their functional properties, such as their emulsifying capacity and solubility [44]. The high temperature and pressure applied during extrusion promote Maillard reactions between proteins and reducing sugars, enhancing the sensory properties of the final product, while reducing anti-nutrients [50,51]. These reactions, along with enzyme inactivation and decreased lipid oxidation, improve the stability and quality of extruded products. Extrusion also effectively reduces the microbial content of food, contributing to food safety and extending the shelf life of food [52].

Particle size plays a critical role in gluten-free flour development, significantly affecting its functional properties [53], and studies have demonstrated that smaller particles enhance the water-holding capacity of flour, improve starch matrix stability during baking, and refine the product’s texture [54].

The main objective of this study is to evaluate the techno-functional and health properties, after extrusion, of a mixture of maize flour with peach peel, at different concentrations, in order to obtain a gluten-free flour formulation with high fiber content and antioxidant properties, as part of a strategy to reduce the negative impact of food waste.

## 2. Materials and Methods

### 2.1. Chemicals

The following chemicals were obtained from Sigma-Aldrich, Co. (St. Louis, MO, USA): α-amylase from porcine pancreas (EC 3.2.1.1), 2,2′-Azinobis 3-ethylbenzothiazoline-6-sulfonic acid (ABTS^•+^), 2,2′-diazobis-(2-aminodinopropane)-dihydrochloride (AAPH), fluorescein, 2,2-diphenyl-1-picrylhydrazyl (DPPH), Folin–Ciocalteu (FC) reagent, gallic acid (GA), and 6-hydroxy-2,5,7,8-tetramethyl-2-carboxylic acid (Trolox), sodium carbonate decahydrate, iron (II) sulfate heptahydrate, iron (III) chloride hexahydrate, 2,4,6-tri(2-pyridyl)-s-triazine (TPTZ), and cellulose. Amyloglucosidase (EC 3.2.1.3) and glucose oxidase–peroxidase (GOPOD) kits were provided by Megazyme International Ireland (Wicklow, Ireland). All the solvents were high-performance liquid chromatography (HPLC) grade (Sigma-Aldrich Co., Madrid, Spain, and Merck KGaA, Darmstadt, Germany).

The chromatographic standards were luteolin, luteoilin-4′O-glucoside, luteolin-7-O-glucoside, catechin, epicatechin, kaempferol-3-O-rutinoside, quercetin-3-O-glucoside, gallic acid, 2,3-dihydroxybenzoic acid, quinic acid, 4-hydroxybenzoic acid, p-coumaric acid, ferulic acid, and apigenin, 3-feruloylquinic acid, quercetin 3-O-rutinoside, procyanidin dimer B, caffeoylquinic acid, cyanidin 3-o-galactoside, cyanidin 3-o-glucoside, and petunidin 3-o-arabinoside, which were obtained from Merck (formerly Sigma-Aldrich, Arklow, Co., Wicklow, Ireland).

### 2.2. Raw Materials

**Figure 1:**
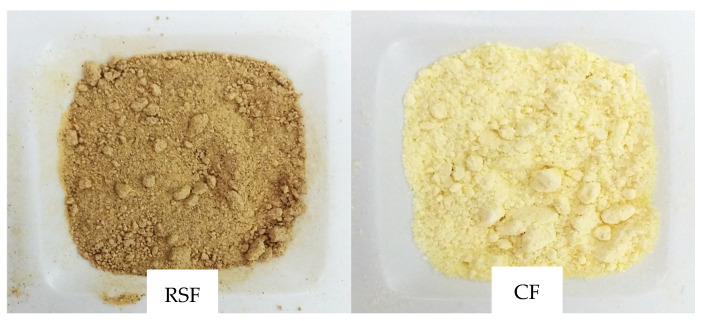
Raw material used for extrusion: Royal Summer flour (RSF) and corn flour (CF).

Peach peel as a by-product and corn (*Zea mays*) flours were used in this study (Figure 1). Corn flour was kindly supplied by a local milling company, Molendum Ingredients (Coreses, Zamora, Spain). Peach peel was supplied by the Institute of Agrifood Research and Technology (Lleida, Spain). The fruits were collected from trees in commercial orchards, located in the Lleida area (Spain).

### 2.3. Extrusion Process

Native (non-extruded) and extruded flours were prepared using dry and milled peach peel and corn flours. The blends were prepared using a ratio of 5:95 and 15:85 (peach peel and corn, respectively). The moisture content of the final mixture was adjusted to 150 g kg^−1^ and maintained overnight at room temperature to encourage homogenous flour hydration. The moisture content was determined by a moisture analyzer (Sartorius mod. MA35, GmbH & Co. KG, Otto-Brenner-Strasse, Germany).

**Figure 2:**
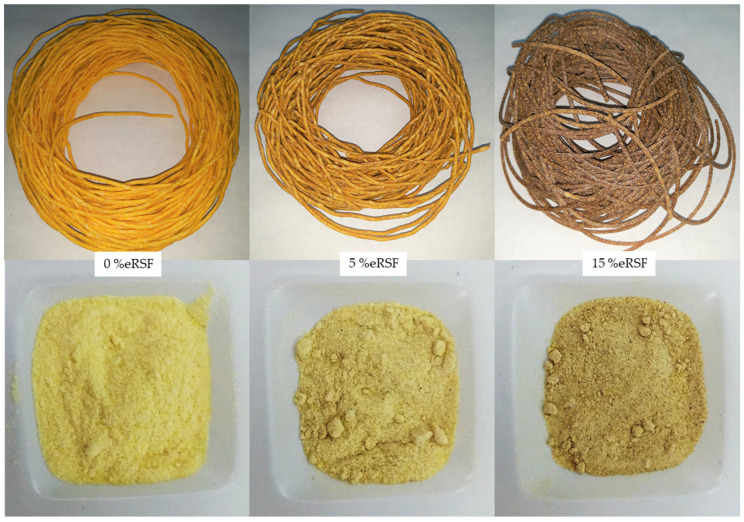
Extruded materials and their corresponding flours: 0% extruded Royal Summer flour (0%eRSF), 5% extruded Royal Summer flour (5%eRSF), and 15% extruded Royal Summer flour (15%eRSF).

Corn and blend flours were extruded using a single-screw lab-scale extruder (Brabender mod.KE19 20 DN, Duisburg, Germany). The screw was 19 mm in diameter, with a length to diameter ratio of 25 (L/D). The feed rate was fixed at 45–150 rpm and the resulting pressure was monitored. The profile temperature of the first three-barrel section was set at 40, 70, and 100 °C, from Section 1 at the feeding point to Section 3, respectively. The temperature at the die/exit of Section 4 was set to 106 °C. The extruded flours (0% extruded Royal Summer flour (0%eRSF), 5% extruded Royal Summer flour (5%eRSF), and 15% extruded Royal Summer flour (15%eRSF)) were dried overnight at room temperature and, afterwards, were milled, using a mill (Model Cyclotec 1093, Foss, Hilleroed, Denmark) fitted with a 0.5 mm screen (Figure 2). The extruded flours were stored in air-tight polyethylene/plastic bags, in dark conditions, at room temperature, until further analyses.

### 2.4. Proximal Composition

The moisture content was measured gravimetrically by drying the samples at 100 °C for 24 h. The total protein content was determined using the Dumas method, the Association of Official Agricultural Chemists (AOAC) method 990.03 [55], in an elemental analyzer. A conversion factor of 5.7 was used to calculate the protein content from the nitrogen values. The total fat content was determined using dried samples, extracted with petroleum ether (BP 40–60 °C), after 4 h in a Soxtec fat extraction unit (AOAC 2005, method 2003.05) [55]. The ash content was determined by sample incineration in a muffle furnace at 550 °C for 5 h (AOAC 2005, method 923.03) [55]. The carbohydrate content was estimated according to the carbohydrates by difference concept. The total dietary fiber (TDF) content was evaluated using a kit provided by Sigma (TDF100A-1KT, St. Louis, MO, USA), in accordance with the manufacturer’s instructions, based on the AOAC method 985.29 [55]. The available starch was measured in the flours, following the protocol described in regard to the total starch assay kit from Megazyme (K-TSTA 03/20, Wicklow, Ireland). All the parameters were evaluated in duplicate. The proximal composition analysis and starch were expressed as g 100 g^−1^ of dry matter (d.m.).

### 2.5. Colorimetric Analysis

The color was measured using a colorimeter (CM-2600d, Konica Minolta Osaka, Japan), with the D65 standard illuminant, a 45/0 sensor, and the 10° standard observer. The instrument was calibrated using the white tile standard (L* = 93.97, a* = −0.88 y b* = 1.21). The color parameters, namely lightness (L*), redness (a*), and yellowness (b*), were expressed in accordance with the CIELAB color space. The measurements were taken from the samples, packaged in transparent plastic bags, at ten different points per sample. To reduce the reflection of the plastic, the specular component was excluded (SCE mode).

### 2.6. Extract Preparation

One gram of the sample was added to 10 mL MeOH/H_2_O (50:50, *v/v*; acidified to pH 2 with 0.1 M HCl) and shaken in the orbital shaker–incubator ‘ES-20’ (Biogen Científica, S.L.) (25 °C, 250 rpm, 20 min), with a ceramic homogenizer. After this, centrifugation was performed in a 5810R centrifuge (Eppendorf) (20 °C, 4000 rpm, 10 min) and the supernatant liquid was removed and retained. A second and third extraction were carried out, following the same procedure, and the newly collected fraction was pooled with the previous one. Then, 10 mL of acetone/H_2_O (70:30, *v/v*) was added to the pellet and shaken in an orbital shaker (25 °C, 250 rpm, 20 min), with a ceramic homogenizer, and the steps described above were repeated. A final extraction with acetone/H_2_O (70:30, *v/v*) occurred. All the fractions from each extraction step were pooled and filtered using Whatman grade 1 cellulose paper. Subsequently, the samples were concentrated in a Multivapor™ P-12 system (Buchi) to a volume of 20 mL.

### 2.7. Total Phenol Content (TPC)

The TPC was measured using the Folin-Ciocalteu method, as described by Slinkard and Singleton [56], with modifications. The absorbance of the sample was measured at 765 nm with a microplate reader (Fluostar Omega, BMG, Ortenberg, Germany). The results were expressed as mg gallic acid equivalent (GAE) per 100 g of the sample, using a calibration curve, with gallic acid used as the standard (100–600 µM). The samples were evaluated in duplicate.

### 2.8. Characterization of Phenolic Compounds Using High-Performance Liquid Chromatography—Electrospray Ionization–Quadrupole Time-of-Flight Mass Spectrometry (HPLC–ESI–QTOF-MS)

Royal Summer peel flour from two different seasons (RSF1 and RSF2) and flour from 15% extruded RSF were analyzed using high-performance liquid chromatography coupled with electrospray ionization–quadrupole time-of-flight mass spectrometry (HPLC–ESI–QTOF-MS). Separation was carried out using the Agilent 1200 HPLC system (Agilent Technologies, Santa Clara, CA, USA), equipped with a diode array detector (DAD, Agilent G1315B) and a QTOF mass analyzer (Agilent G6530A), using atmospheric pressure electrospray ionization (ESI). A Luna C18 column (250 mm × 2 mm i.d., 5 μm, Phenomenex, Torrance, CA, USA), maintained at 25 °C, was employed. The gradient elution utilized 0.1% aqueous formic acid (solvent A) and 0.1% formic acid in acetonitrile (solvent B), with a flow rate of 0.4 mL/min. The gradient profile was as follows: 8% solvent B at 0 min, increasing to 23% at 10 min, 50% at 15 min, the remaining 50% until 20 min, reaching 100% at 23 min, followed by re-equilibration. An injection volume of 2 μL was used. Data acquisition was performed in negative ion mode over a mass range of 100–1200 Da, with a source temperature of 325 °C and a gas flow rate of 10 L/h. Compound identification was based on the retention time of the commercial standards, when available. For unidentified compounds, the molecular formulas proposed by the MassHunter Workstation software version 4.0 were compared with the reported phenolic compounds in cereals, with a maximum error tolerance of 10 ppm. MS/MS experiments were conducted using the auto MS/MS acquisition mode and the observed fragmentation patterns were matched against the reported phenolic compound fragments.

Quantification of the phenolic compounds was achieved using calibration curves of the authentic standards, including gallic acid, apigenin, ferulic acid, (-)-epicatechin, kaempferol, sinapic acid, *p*-coumaric acid, avenanthramide C, and secoisolariciresinol, at concentrations ranging from 0.1 to 25 μg mL^−1^, demonstrating good linearity (R^2^ > 0.99). The results were expressed as the mean ± standard deviation of two independent replicates, reported in mg 100 g^−1^ d.m.

### 2.9. Total Antioxidant Capacity (TAC)

The total antioxidant capacity (TAC) was determined in regard to extracts using the 2,2-diphenyl-1-picrylhydrazyl radical (DPPH^•^), 2,20-azinobis-(3-ethylbenzothiazoline-6-sulphonate (ABTS^∙+^), the oxygen radical absorbance capacity (ORAC), and ferric-reducing capacity potential (FRAP) assays. Additionally, modified DPPH^•^ and ABTS^∙+^ methods were used on solid samples without prior extraction (Q-DPPH^•^ and Q-ABTS^∙+^), for the evaluation of the total antioxidant activity of the samples. All the analyses were performed in duplicate.

#### 2.9.1. DPPH^•^ Radical Scavenging Activity (DPPH^•^)

The DPPH^•^ was analyzed using the method described by Brand-Williams et al. [57], with some modifications. Specifically, 250 µL of the sample extract, Milli-Q water, and 120 µM DPPH^•^ methanol solution were mixed in a ratio of 1:4:5, *v:v:v*. Absorbance was measured at 525 nm after 30 min, using a Spectrostar Omega microplate reader (BMG Labtech, Ortenberg, Germany). The Trolox solution (7.5–210 µM) standard curve was used to calculate the DPPH^•^ values and were expressed as µmol TE per 100 g d.m.

#### 2.9.2. ABTS^•+^ Radical Cation Scavenging Activity (ABTS^•+^)

The ABTS^•+^ assay was conducted following the method described by Re et al. [58], as adapted by Martin-Diana et al. [59]. A stock solution was prepared by mixing 7 mM ABTS^•+^ and 2.45 mM potassium persulphate in a 1:1 ratio (v:v) and left in the dark at room temperature, overnight. A working solution was then prepared by diluting the stock solution to an absorbance of 0.7 ± 0.02 at 730 nm, using 75 mM phosphate buffer (pH 7.4), and equilibrated at 30 °C.

Next, 20 µL of the extracts, blank, or Trolox standard (7.5–210 µM), and 200 µL of the ABTS^•+^ working solution, were added to a 96-well microplate. After incubating in darkness for 1 h, the absorbance was measured at 730 nm, using a Spectrostar Omega microplate reader (BMG Labtech, Ortenberg, Germany). The ABTS^•+^ results were expressed as µmol TE per 100 g d.m.

#### 2.9.3. Oxygen Radical Absorbance Capacity (ORAC)

The ORAC method was carried out, following the procedure previously described by Ou et al. [60], with minor modifications. The Trolox standard curve (7.5–210 µM) and the samples were diluted in phosphate buffer (75 mM, pH 7.4). Fluorescence was measured using a microplate reader (CLARIOstar Plus, BMG, Ortenberg, Germany), with the excitation and emission filters set at 485 nm and 520 nm, respectively, over a 150 min period, at 37 °C.

To determine the results, the areas under the fluorescein decay curves, comparing the blank and the sample, were calculated and expressed as µmol TE per 100 g d.m.

#### 2.9.4. Ferric-Reducing Antioxidant Power (FRAP)

The FRAP assay was conducted following the methodology outlined by Benzie and Strain [61]. The FRAP working solution was prepared by combining 300 mM acetate buffer (pH 3.6), 10 mM TPTZ dissolved in 40 mM hydrochloric acid, and 20 mM ferric chloride hexahydrate solution, with a volume ratio of 10:1:1, respectively.

The reaction was carried out by mixing 20 µL of the sample, blank, or standard, with 1.9 mL of the FRAP working solution. Distilled water and the ferrous sulphate heptahydrate solution (400–3000 µM) were used as the blank and standard, respectively. Absorbance was measured at 593 nm, using a Spectrostar Omega microplate reader (BMG Labtech, Ortenberg, Germany). The results were expressed as µmol of Fe^2+^ equivalent per 100 g of d.m.

#### 2.9.5. Q-DPPH^•^ Radical Scavenging Activity and Q-ABTS^•+^ Radical Cation Scavenging Activity

For the analysis of the solid samples, the Q-DPPH^•^ method was used, following the protocol described by Serpen et al. [62], with some modifications. Ten milligrams of the powdered solid sample (<300 µm) was weighed and mixed with 30 mL of DPPH• working solution (60 µM), prepared in methanol. The samples were incubated for 30 min at 700 rpm, using a Thermomixer Compact (Eppendorf, AG, Hamburg, Germany). Following incubation, the samples were centrifuged at 14,000× *g* for 2 min and the absorbance was measured at 515 nm. The Trolox calibration curve (7.5–210 µM) was used as the standard. The results were reported as µmol TE per 100 g d.m.

For the Q-ABTS^•+^ method, the protocol described by Serpen et al. [62], as modified by Martin-Diana et al. [59], was followed. Ten milligrams of the powdered sample was mixed with 30 mL of ABTS^•+^ working solution, prepared as outlined in Section 2.9.2. Additionally, 3 mL of a methanol/water mixture (50:50, v:v) was added to the samples, to match the final volume of the standard curve. The Trolox calibration curve (7.5–210 µM) served as the standard. After 30 min of incubation in the dark, the absorbance was measured at 730 nm, and the results were expressed as µmol TE per 100 g d.m.

### 2.10. Glycemic Index (GI)

Before the determination of the glycemic index (GI) was carried out in regard to the flours, the available starch in the samples was first evaluated, using a total starch assay kit from Megazyme (K-TSTA 03/20, Wicklow, Ireland). Afterwards, the in vitro starch hydrolysis rate was determined as described by Gularte and Rosell [63], with slight modifications. Samples containing 50 mg of available starch were dissolved in 2 mL Tris-maleate buffer (0.1 M, pH = 6) and held at 37 °C for 1 min, with constant stirring. Then, 2 mL of an enzymatic solution, containing porcine pancreatic α-amylase (460 U/mL) and amyloglucosidase (6.6 U/mL), was added. Aliquots of 150 µL were taken at times of 0, 10, 20, 30, 60, 90, and 120 min, during the incubation period. These aliquots were immediately placed in a bath of boiling water for 5 min to stop the enzymatic reaction and were then cooled in ice. Then, 150 µL of absolute ethanol was added and the sample was centrifuged (4 °C, 10,000× *g* for 5 min). The pellet was washed with 150 µL ethanol/water (1:1, *v/v*) and the supernatants were pooled together and stored at 4 °C for the subsequent colorimetric analysis of the reducing sugars, using a GOPOD kit (Megazyme, Bray, Ireland). The hydrolysis index (HI) and predicted glycemic index (pGI) values were calculated using the formulas proposed by Granfeldt [64].

### 2.11. Scanning Electron Microscopy (SEM)

Scanning electron microscopy was carried out using a scanning electronic microscope (FEI QUANTA 200, Graz, Austria). Images were taken using 100×, 1500×, 3000×, and 6000× magnifications of the surface and section. A voltage of 5.00 keV was used and the spot size was 3.0.

### 2.12. Functional Properties

The water-holding capacity (WHC) and oil-holding capacity (OHC) and bulk density (BD) were determined in accordance with the procedure reported by Garcia-Vaquero et al. [65]. The WHC and OHC were expressed as the weight of water or sunflower oil in grams per gram of solid. The foaming capacity (FC) was measured as the volume of foam generated as a percentage of the initial volume, as described by Lafarga et al. [66]. The emulsifying capacity (EC) of the samples was determined as described by Lafarga et al. [66], indicating the capacity of the sample to aid emulsion formation. The least gelling concentration (LGC) of the samples was determined according to the method proposed by Sathe and Salunkhe [67]. The LGC was estimated as the critical concentration below which no self-supporting gel was formed. The swelling capacity (SC) was measured following the method described by Yadav and colleagues [68].

### 2.13. Statistical Analysis

The data are presented as the mean ± standard deviation from at least three independent experiments. An analysis of variance (ANOVA), followed by Duncan’s post hoc test, were conducted to identify significant differences between the mean values. All the statistical analyses, except for the quantification of the phenolic compounds using HPLC–ESI–QTOF-MS, were performed using Statgraphics Centurion XVI^®^ (StatPoint Technologies, Inc., Warrenton, VA, USA).

The data on the phenolic compounds quantified via HPLC–ESI–QTOF-MS were analyzed using IBM SPSS Statistics 28.0. The normality of the data was assessed using the Shapiro–Wilk test. As the data did not conform to normality, the Kruskal–Wallis test was applied, followed by Mann–Whitney U tests, to carry out comparisons between the independent groups. The results are expressed as mean values, with their corresponding standard deviations. Statistical significance was defined as a *p*-value < 0.05.

## 3. Results and Discussion

### 3.1. Proximal Composition

The proximal composition analysis showed the impact of extrusion and RSF incorporation on the nutritional properties of the final flours. Table 1 shows the proximal composition of the native flours (RSF and CF) and extruded flours (0%eRSF, 5%eRSF, and 15%eRSF). The ash content of corn flour (CF) was significantly (*p* < 0.05) lower than Royal Summer peel flour (RSF) and the extruded flours. The values obtained ranged from 0.41 to 3.70 g 100 g^−1^, with an increase in the ash content of the extruded samples when the percentage of Royal Summer peel flour added was increased.

The ash content highlights the mineral-rich nature of RSF compared to CF, with significant increases in the extruded samples as the RSF concentration increases. This increase demonstrates the potential of RSF to enhance the mineral content in functional food formulations.

Extrusion significantly increased the fat content, which ranged from 0.47 to 0.80 g 100 g^−1^ in native flours (RSF and CF, respectively), increasing to 0.77–1.71 g 100 g^−1^ after extrusion. Similarly, the fat content increased significantly post-extrusion, likely due to the release of lipids during the high-temperature and pressure conditions of the process, which disrupts cellular structures. These results indicated that RSF and extrusion synergistically improved the nutritional profile of the flours in terms of the mineral and fat content.

The extrusion process and the incorporation of RSF significantly reduced (*p* < 0.05) the moisture content of the CF. The moisture values obtained were 5.44 and 13.22 g 100 g^−1^ in native RSF and CF flours, respectively, where in the case of the extruded flours, it ranged from 8.79 to 9.17 g 100 g^−1^. The decrease in the moisture content reflects the dehydration effect of the extrusion process. This reduction enhances the shelf life and stability of flour, although it is slightly mitigated by the RSF addition, which may retain water due to its high fiber content.

The protein levels remained consistent across all the samples, suggesting that neither extrusion nor RSF incorporation affected the protein integrity of the flour. The values ranged from 6.56 to 6.88 g 100 g^−1^. The carbohydrate content showed significant differences (*p* < 0.05) between the native flours, with a value of 83.51 for the RSF and 79.01 for the CF, while for the extruded flours no significant differences were obtained (*p* < 0.05), achieving values from 82.00 to 83.02 g 100 g^−1^.

The fiber content of the native flours was 22.50 and 4.30 g 100 g^−1^ for the RSF and CF, respectively. In extruded flours, the fiber content increased from 2.10 to 5.90 g 100 g^−1^, with a clear positive effect on the fiber content due to RSF addition. The extruded flour with 15% RSF may be suitable for ‘source of fiber’ labeling, according to EU Regulation (EC) No 1924/2006 [69], a nutritional claim for products with more than 3 g fiber per 100 g.

The formulation had a significant effect on the starch content (*p* < 0.05), with lower values in the 5% and 15% RSF samples (63.42 and 67.40 g 100 g^−1^, respectively), as compared to the 0% RSF flour (77.76 g 100 g^−1^).

The fiber content increase, along with the reduction in starch content, in RSF-enriched flours, supports their suitability for health-oriented food applications, offering lower glycemic potential and enhanced dietary-fiber benefits. Overall, RSF addition and extrusion demonstrate complementary effects, creating value-added flours, with improved functional and nutritional characteristics.

### 3.2. Colorimetric Analysis

The color of native and extruded flours was determined with a CIELAB scale and the results are shown in Table 2. The colorimetric analysis of the native and extruded flours reveals significant effects of RSF addition and extrusion on the flours’ visual attributes, which are critical for consumer acceptance and product characterization.

The lightness of the extruded flours showed significant differences (*p* < 0.05), decreasing from 84.81 to 74.48 when the RSF percentage increased. This is due to Royal Summer peel flour having a lightness value of 63.99 compared to the CF value of 85.65, reducing the lightness value to 12.18% in the extruded flours. The decrease in lightness (L*) values with the increasing RSF concentration indicates that the darker pigment of RSF, originating from the peach peel, strongly influences the overall color of the blended flours. This reduction in lightness may also be attributed to the Maillard reaction during extrusion, where sugars and amino acids react in response to heat, producing browning compounds that further darken the product. Such changes can be strategically used to enhance the visual appeal of darker colored food products or functional blends.

The color parameter a* (green to red) showed a value of 10.46 for the RSF and 4.09 for the CF. The extruded flours showed significant differences between them, increasing the value of the a* parameter, due to the color provided by the peach peel, from 3.37 to 3.92. The increase in the a* parameter with the RSF addition highlights the contribution of the red pigments in the peach peel, likely anthocyanins or carotenoids, which intensify the reddish hue of the extruded flours. The slight increase in the a* parameter after extrusion suggests that thermal processing enhances the visibility of these pigments, potentially due to pigment release or the breakdown of cellular structures.

The values obtained for the color parameter b* (blue to yellow) were 14.61 for the RSF and 22.41 for the CF. Significant differences (*p* < 0.05) were observed between the values obtained for the extruded flours, which decreased from 24.86 to 19.54 when the percentage of RSF increased. The reduction in the b* parameter with a higher amount of RSF incorporation suggests a dilution of the yellow color from corn flour by RSF, which is less yellow. Additionally, extrusion itself might degrade carotenoid pigments that are naturally present in CF, contributing to the observed changes in the yellow–blue axis. These color modifications are crucial for tailoring flours to specific product formulations, particularly in food industries focusing on natural ingredients and visually distinct offerings. Similar to lightness and color parameter a*, the incorporation of RSF and the extrusion process produces changes in the color of the native flour. 

### 3.3. Total Phenol Content (TPC)

**Figure 3:**
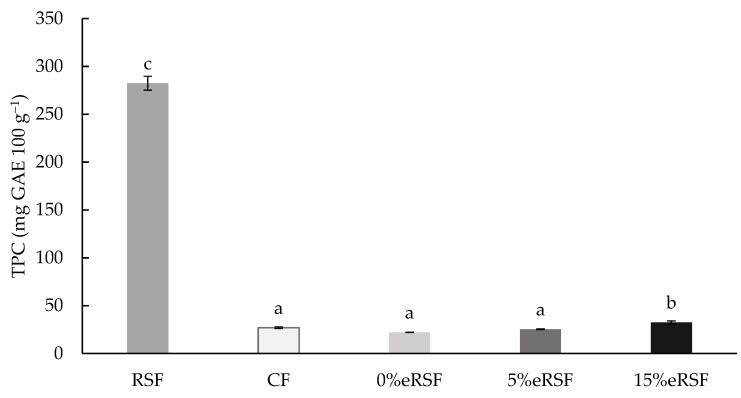
Total phenol content (TPC, mg GAE 100 g^−1^ of flour) of native and extruded flours. Abbreviations: RSF, Royal Summer flour; CF, corn flour; 0 %eRSF, 0% extruded Royal Summer flour; 5 %eRSF, 5% extruded Royal Summer flour; 15 %eRSF, 15% extruded Royal Summer flour. Different lowercase letters indicate significant differences in the mean values (one-way ANOVA, Duncan’s test, *p* ≤ 0.05) between the samples.

The total phenol content (TPC) was determined in the native and extruded flours (Figure 3). The values ranged from 22.11 to 282.47 mg GAE 100 g^−1^, showing significant differences (*p* < 0.05) between the samples. Royal Summer peel flour (RSF) was the sample with the highest TPC content. In addition, it was observed that the incorporation of 15% RSF during the extrusion process significantly increased (*p* < 0.05) the TPC compared to the extruded flour without RSF (0 %eRSF), from 22.11 to 32.49 mg GAE 100 g^−1^.

The evaluation of the TPC in the native and extruded flours showed the significant advantage of the incorporation of Royal Summer peel flour (RSF) regarding the phenolic profile of the flour. The exceptionally high TPC in RSF, reaching 282.47 mg GAE 100 g^−1^, underscores its potential as a rich source of phenolic compounds. These bioactive molecules contribute to antioxidant activity, which is linked to reducing oxidative stress and associated chronic diseases [70]. The significant increase in TPC observed in extruded flours with 15% RSF incorporation, increasing from 22.11 mg GAE 100 g^−1^ in 0% RSF to 32.49 mg GAE 100 g^−1^, highlights the additive and synergistic effects of RSF inclusion during extrusion processing. Extrusion technology influences the bioavailability of phenolic compounds through structural modification and thermal effects. The high temperature and mechanical shear applied during extrusion can disrupt plant cell walls, releasing bound phenolics and enhancing their extractability [71]. However, thermal degradation of some phenolics may occur, which could explain the moderate TPC values in the extruded flours compared to the native RSF. This balance underscores the importance of optimizing the extrusion conditions to maximize phenolic retention. The incorporation of RSF in extruded flours not only enriched their antioxidant capacity, but also meets the increasing consumer demand for functional foods with health-promoting properties, demonstrating its applicability in the development of nutritionally enhanced food products.

### 3.4. HPLC–ESI–QTOF-MS

The phenolic profile determined by HPLC–ESI–QTOF-MS in RSF and extruded flour with 15% RSF, highlights the variability in the phenolic compound composition and concentration among the samples. Table 3 shows the values obtained in regard to the phenolic profile, determined by HPLC–ESI–QTOF-MS, of the Royal Summer peel flour obtained in two different seasons (RSF1 and RSF2) and of the extruded flour with 15% RSF (15%eRSF). Differences were observed in the phenolic profile of the analyzed samples, where five compounds were identified in RSF1 and the content reached 73.6 mg 100 g^−1^, while for RSF2, eight compounds were identified and the content reached 66.6 mg 100 g^−1^. However, in the 15%eRSF sample, four compounds were identified and the content was 1.8 mg 100 g^−1^.

RSF1 and RSF2, representing different harvest seasons, showed differences in the type and concentration of the phenolic compounds analyzed. RSF1 contained five major phenolics that were identified, with a total content of 73.6 mg 100 g^−1^, while RSF2 exhibited eight major compounds, with a slightly lower total content of 66.6 mg 100 g^−1^. This seasonal variability may be attributed to environmental factors, such as temperature, sunlight, and soil conditions, which are known to influence the biosynthesis of phenolic compounds in plants [72].

The significantly lower total phenolic content (1.8 mg 100 g^−1^) in the 15%eRSF sample compared to the native RSF suggests a degradation or transformation of the phenolic compounds during the extrusion process. The high temperatures and mechanical shear associated with extrusion are known to degrade certain thermolabile phenolic compounds or cause polymerization and complex formation, reducing their detectability [45]. The reduction in cyanidin derivatives and catechins in the extruded sample could be associated with their susceptibility to heat-induced degradation. Despite this loss, the retention of specific phenolics, such as 3-feruloylquinic acid, in the 15%eRSF sample underscores the differential stability of phenolic compounds in extrusion conditions. The variability in the phenolic stability highlights the importance of optimizing the processing parameters to retain bioactive components, thereby enhancing the functional and nutritional value of extruded products.

### 3.5. Total Antioxidant Capacity (TAC)

The TAC of the native and extruded flours was determined against different free radicals (DPPH^•^, ABTS^∙+^, ORAC, and FRAP). The TAC of the native and extruded flours shows the complex impact of extrusion and the inclusion of RSF on the antioxidant properties of these formulations.

**Figure 4:**
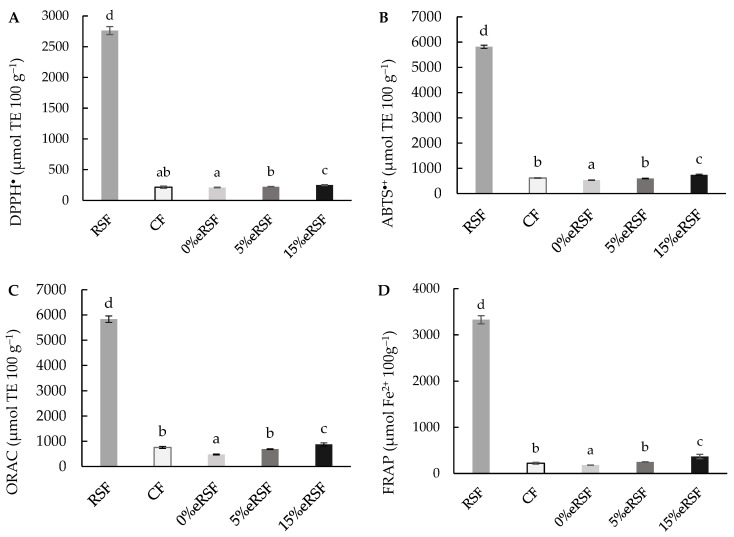
Total antioxidant activity in regard to DPPH (**A**), ABTS^∙+^ (**B**), ORAC (**C**) (µmol Trolox equivalent (TE) per 100 g of flour), and FRAP (**D**) (mmol Fe^2+^ per 100 g of flour). Abbreviations: RSF, Royal Summer flour; CF, corn flour; 0 %eRSF, 0% extruded Royal Summer flour; 5 %eRSF, 5% extruded Royal Summer flour; 15 %eRSF: 15% extruded Royal Summer flour. Different lowercase letters indicate significant differences in the mean values (one-way ANOVA, Duncan’s test, *p* ≤ 0.05) between the samples.

The extrusion process reduced the antioxidant capacity against the DPPH^•^ radical in corn flour, from 215.30 to 210.58 µmol TE 100 g^−1^ (Figure 4A). Reductions in the antioxidant capacity values after extrusion have been consistently reported in regard to different matrices [73,74,75]. Alonso et al. [76] found similar reductions in the TAC during extrusion, which are consistent with findings in other studies, where heat and shear forces lead to the degradation of thermolabile phenolic compounds.

However, the incorporation of RSF in regard to the extrusion significantly (*p* < 0.05) increased the antioxidant activity against DPPH, from 210.58 to 249.60 µmol TE 100 g^−1^. This is due to the higher antioxidant activity of RSF against this radical (2762.70 µmol TE 100 g^−1^).

Similarly, the ABTS^∙+^ radical scavenging activity showed significant differences (*p* < 0.05) related to the extrusion process (Figure 4B). The values obtained in regard to the extruded flours ranged from 532.69 to 745.33 µmol TE 100 g^−1^. The increment in the RSF percentage in regard to the extrusion increased the antioxidant capacity against the ABTS^∙+^ radical to 85,816.22 µmol TE 100 g^−1^. This could be associated with its rich phenolic composition, including compounds with strong radical scavenging properties, such as cyanidin derivatives and caffeoylquinic acids.

The results of the antioxidant capacity determined by the ORAC assay (Figure 4C) showed a significant increase (*p* < 0.05) in the activity after the extrusion process. The values of the extruded flours ranged from 476.00 to 875.71 µmol TE 100 g^−1^, increasing after the addition of RSF in the formulation. The values for the native flours were 5832.07 and 759.25 µmol TE 100 g^−1^ for the RSF and CF, respectively. This enhancement aligns with previous studies showing that RSF-derived phenolics retain their activity under oxidative stress conditions simulated by the ORAC assay [77].

Also, the reducing power (FRAP) of the native and extruded flours was determined (Figure 4D). The value obtained for the reducing power in the native flours was 3327.40 µmol Fe^2+^ 100 g^−1^ for the RSF and 221.47 µmol Fe^2+^ 100 g^−1^ for the CF. Similarly, the incorporation of RSF during extrusion increased the reducing power, from 181.38 to 367.57 µmol Fe^2+^ 100 g^−1^, with the values being higher in the 15%eRSF flour.

**Figure 5:**
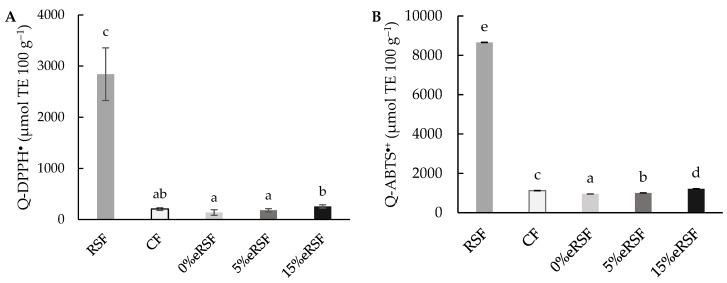
Direct antioxidant properties in regard to Q-DPPH^•^ (**A**) and Q-ABTS^∙+^ (**B**) (µmol Trolox equivalent (TE) per 100 g of flour). Abbreviations: RSF, Royal Summer flour; CF, corn flour; 0%eRSF, 0% extruded Royal Summer flour; 5%eRSF, 5% extruded Royal Summer flour; 15%eRSF, 15% extruded Royal Summer flour. Different lowercase letters indicate significant differences in the mean values (one-way ANOVA, Duncan’s test, *p* ≤ 0.05) between the samples.

In addition, the antioxidant activity in the native and extruded flours was evaluated using direct methods (Q-DPPH^•^ and Q-ABTS^∙+^) and the results obtained are shown in Figure 5. The values obtained for Q-DPPH^•^ were similar to those obtained in the assay with the extracts (DPPH^•^), achieving values of 2840.68 µmol TE 100 g^−1^ for the RSF and 206.02 µmol TE 100 g^−1^ for the CF, while the extruded flours showed an increase in the antioxidant activity, from 138.68 to 255.88 µmol TE 100 g^−1^, according to the increase in the RSF percentage added. However, the results for Q-ABTS^∙+^ were higher than the results obtained in the ABTS^∙+^ assay, with values of 8654.83 µmol TE 100 g^−1^ for the RSF and 1122.21 µmol TE 100 g^−1^ for the CF. The extrusion process reduced the antioxidant capacity of CF to 954. 60 µmol TE 100 g^−1^ (0%eRSF), but after the addition of RSF, an increase in the antioxidant capacity was observed, with values of 1007.23 µmol TE 100 g^−1^ (5%eRSF) and 1220.40 µmol TE 100 g^−1^ (15%eRSF).

The improvement in the TAC with the RSF addition is consistent with reports that phenolic-rich food by-products can enhance the nutritional and functional properties of processed foods [78]. Furthermore, the extrusion process may facilitate the release of bound phenolics from the food matrix, partially offsetting thermal degradation and contributing to the observed increases in the antioxidant capacity in regard to some assays [79]. These findings underscore the potential of incorporating RSF as a functional ingredient to produce antioxidant-enriched flours, addressing the consumer demand for health-promoting food products.

### 3.6. Functional Properties

The extrusion process significantly influences the functional properties of flours, primarily through alterations in the starch structure, as noted by Huang et al. [80]. Table 4 presents the functional properties of the native and extruded flours, demonstrating notable changes post-extrusion. The apparent density (AD) values increased significantly (*p* < 0.05) following extrusion, ranging from 0.54 to 0.91 g/mL. This increase can be attributed to starch gelatinization and molecular rearrangement during the extrusion process, which compacts flour particles, as supported by the findings of Zhang et al. [81].

The oil-holding capacity (OHC) values ranged from 3.31 to 3.98 mL/g, with no significant differences (*p* < 0.05) observed between the flours. This consistency may indicate that extrusion has a limited impact on the hydrophobic interactions of proteins and lipids responsible for oil retention, aligning with the observations by Enobong and Madu [82].

In contrast, the water-holding capacity (WHC) showed significant variation. The WHC of the RSF was notably higher (6.44 mL/g) compared to the CF (2.98 mL/g). While extrusion improved the WHC of corn flour to 5.48 mL/g, the incorporation of RSF during extrusion reduced the WHC by 30–33%, with the lowest value (3.64 mL/g) observed in the 15%eRSF flour. This reduction may be the result of the competitive binding of water between the starch and fiber components, as described by Arribas et al. [83].

The swelling capacity (SC) was only detected in the 15%eRSF flour (2.94 mL/g), likely due to the higher proportion of extruded material enhancing its water absorption and swelling properties. This behavior aligns with previous studies highlighting the influence of extrusion on the expansion and water absorption properties of starch-rich flours [84].

The emulsifying capacity (EC) ranged from 1.85% to 20.99%, with RSF showing the highest value. However, extrusion significantly reduced the EC of the flours and the 15%eRSF exhibited no emulsifying capacity. This decline may be attributed to protein denaturation and the reduced surface activity of the protein molecules during extrusion, as previously reported by Gao et al. [85].

The foaming capacity (FC) remained constant (50%) in the native flours, but was absent in all the extruded flours. The thermal and mechanical stresses during extrusion likely denaturize the protein structures necessary for foam formation, consistent with the findings of Alam et al. [86].

The gelation capacity (GC) varied between the flours, with the RSF showing higher values (20.05%) compared to the CF (8.05%). While extrusion did not significantly affect the GC, higher RSF incorporation (15%) improved the GC to 12.12%. This improvement is consistent with the increased gel-forming ability of fiber-enriched flours in extrusion conditions, as noted by Huth et al. [87].

The changes in these functional properties highlight the potential of extrusion technology to tailor flour properties for specific applications. The enhanced WHC, SC, and GC in RSF-enriched flours demonstrate their potential use in gluten-free products and functional food formulations. However, the reduction in the EC and FC may limit their suitability in regard to applications requiring emulsification or foaming properties. Further research is warranted to optimize the extrusion parameters to balance these functional properties effectively.

### 3.7. Glycemic Index

**Figure 6:**
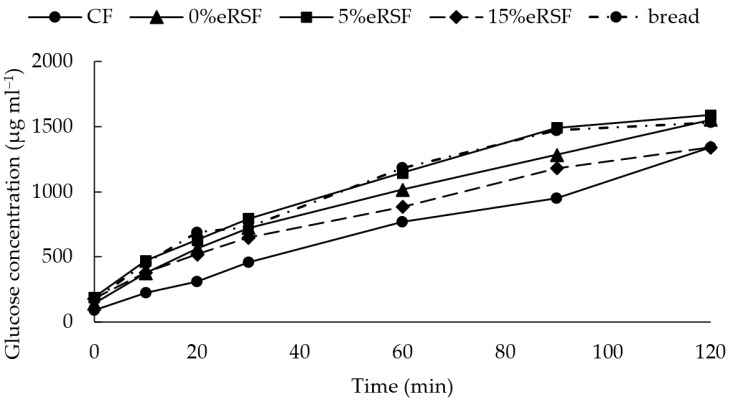
Glucose kinetics related to consumption (µg mL^−1^) in terms of native and extruded flours. Abbreviations: CF, corn flour; 0%eRSF, 0% extruded Royal Summer flour; 5%eRSF, 5% extruded Royal Summer flour; 15%eRSF, 15% extruded Royal Summer flour.

The starch hydrolysis kinetics were analyzed, the hydrolysis index (HI) was calculated, and the glycemic index (GI) of the native and extruded flours was estimated (Figure 6, Table 5). The starch hydrolysis kinetics of the extruded flour containing 5% RSF were comparable to those of the white bread used as a control, with no significant difference observed between their respective GI values. In contrast, the 0%eRSF flour exhibited slower glucose release, resulting in a GI of 86.04.

However, the CF and 15%eRSF flours showed lower starch hydrolysis kinetics and, therefore, a lower GI, with a value of 65.90 for the CF and 78.09 for the 15%eRSF. This indicates that the incorporation of RSF into the formulation of extruded flours reduces the GI.

### 3.8. Scanning Electron Microscopy (SEM)

**Figure 7:**
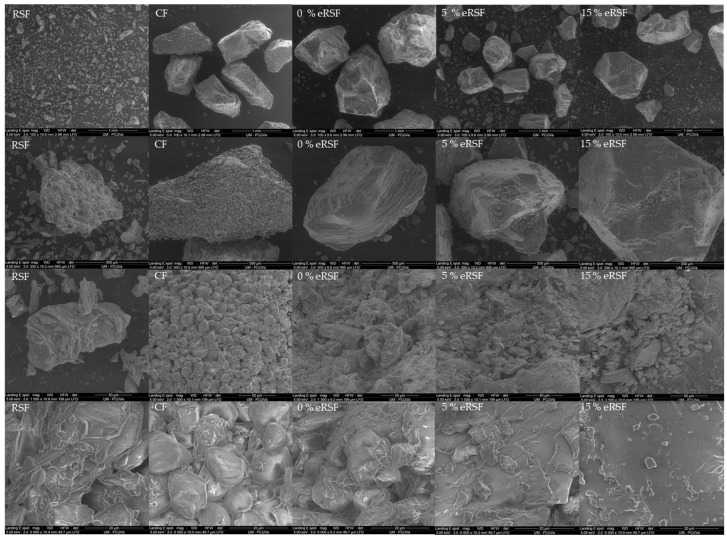
SEM micrographs of the surface of the raw materials used for extrusion (Royal Summer flour (RSF) and corn flour (CF)) and the extruded flours (0% extruded Royal Summer flour (0%eRSF), 5% extruded Royal Summer flour (5%eRSF), and 15% extruded Royal Summer flour (15%eRSF)).

Figure 7 shows the SEM images used to assess the microstructure of the native and extruded flours. The characteristic morphology of the starch granules was evident in the native flours (RSF and CF). In the RSF, the granules exhibited an irregular and poorly defined structure, surrounded by cellular material, such as fiber and protein fractions. Similarly, the maize starch granules displayed irregular shapes with polyhedral faces, consistent with the observations by Jalali et al. [88].

The inclusion of RSF appeared to smooth the microstructure of the extruded samples, an effect comparable to that observed when carrot pomace was used as a fiber-enrichment ingredient in corn extruded flours [89], which also had enhanced expansion properties. These structural changes underscore the significant influence of both composition and extrusion processing on the functional properties of flour within food systems.

The bulk density (BD) of the flours increased markedly post-extrusion, with the 0%eRSF sample exhibiting the highest value (0.91 g mL^−1^), indicating a more compact matrix, advantageous for packaging and storage. The oil-holding capacity (OHC) remained stable across all the samples, ranging from 3.31 mL g^−1^ to 3.98 mL g^−1^, suggesting that the extrusion process did not significantly alter the flour’s capacity to retain oil. In contrast, the water-holding capacity (WHC) showed a notable decline following extrusion, with the RSF recording the highest WHC value (6.44 mL g^−1^) among the native flours. Post-extrusion, the WHC decreased to 3.82 mL g^−1^ and 3.64 mL g^−1^ for the 5%eRSF and 15%eRSF, respectively, likely due to structural changes in the starch and protein denaturation, which reduce the water retention capacity.

Swelling capacity (SC) was observed exclusively in the 15%eRSF sample (2.94 mL g^−1^), which may be attributed to the higher proportion of extruded material, enhancing its water absorption and swelling potential. The emulsifying capacity (EC) was highest in the RSF (20.99%), highlighting its potential for applications in emulsion-based products, such as dressings. However, extrusion significantly diminished the EC, with the 15%eRSF sample exhibiting no measurable emulsifying ability, likely due to the denaturation of proteins critical for emulsification.

The foaming capacity (FC) remained consistent in the native flours (RSF and CF), at 50%, but was absent in the extruded samples, further corroborating the adverse effects of extrusion on foaming properties. The least gelation concentration (LGC) was higher in the RSF (20.05%) compared to the CF (8.05%), reflecting RSF’s weaker gel-forming potential. Interestingly, extrusion enhanced the gelation capacity, with the 15%eRSF sample showing the highest LGC (12.12%), potentially due to the improved solubility and gelling properties of starch and proteins post-extrusion.

These findings demonstrate the trade-offs associated with extrusion processing. While extrusion can improve properties, such as the bulk density and gelation capacity, it may negatively affect other functional attributes like emulsification and foaming. Such insights are invaluable for optimizing flour formulations and selecting processing methods for specific food applications.

In line with our findings, previous studies have highlighted the significant effects of extrusion on the functional properties of flours, including emulsifying, foaming, and water retention capabilities. For example, extrusion has been shown to enhance the water-holding capacity (WHC) in certain proteins, as demonstrated by a study on rice protein, where extrusion improved the WHC by up to 37.74% in optimal conditions (200 rpm, 130 °C, and 25% moisture) [85]. Similarly, emulsifying capacity (EC) can also be positively impacted by extrusion, with one study reporting a 152.82% improvement in emulsion stability following the extrusion of rice protein. These results align with our findings, where extrusion decreased the emulsifying and foaming properties in the 15%eRSF, likely due to protein denaturation, which disrupts the emulsifying interfaces necessary for stabilization [90].

Additionally, structural alterations that occur during extrusion, such as modifications in protein conformation (e.g., an increase in α-helix structures), can influence the texture and gelling characteristics of proteins [85]. This aligns with our findings, where extrusion enhanced the least gelation concentration (LGC) in RSF and its extruded variants. Previous research has also highlighted that extrusion promotes gelation, by improving the solubility and gelling potential of both starch and proteins [90].

Taken together, these studies corroborate our results, indicating that while extrusion improves certain properties, such as gelation and density, it concurrently diminishes emulsifying and foaming abilities. This underscores the trade-offs inherent in extrusion processing.

These observations emphasize the importance of understanding the structural and functional effects of extrusion in order to optimize food formulations that balance the desired textural, emulsifying, and gelling properties.

## 4. Conclusions

This study demonstrated that incorporating Royal Summer peach peel flour into gluten-free flour formulations can significantly enhance their nutritional and functional properties. The extrusion process was fundamental in transforming these mixtures, contributing to improved dietary fiber content, antioxidant capacity, and certain functional properties, such as gelation and swelling capacities. These changes underscore the potential of RSF as a sustainable ingredient for developing health-promoting gluten-free products.

The RSF-enriched flours showed a remarkable increase in dietary fiber content, particularly in the 15% RSF formulation, which achieved compliance with the “source of fiber” labelling regulations. The inclusion of RSF also enhanced the antioxidant activity across multiple assays, highlighting the contribution of phenolic compounds derived from peach peel. These bioactive components provide additional health benefits by reducing oxidative stress and promoting overall wellness.

In terms of functional properties, the addition of RSF demonstrated both benefits and trade-offs. While the bulk density and gelation capacity were enhanced, properties such as the emulsifying and foaming capacities were adversely affected. The incorporation of RSF also reduced the glycemic index of the flours, making them a viable option for individuals with diabetes or those seeking low-glycemic alternatives.

The findings highlight the dual benefit of RSF as both a valorized food by-product and a functional ingredient. Its incorporation aligns with global sustainability goals by addressing food waste and offering innovative solutions for health-oriented food products. Future research should focus on optimizing the extrusion parameters to balance the functional properties of flour and further explore the scalability of this approach in terms of industrial applications.

The study underscores the potential for RSF-enriched flours to meet the growing demand for sustainable, functional, and health-promoting food products, making a significant contribution to both environmental sustainability and nutritional innovation.

## Figures and Tables

**Table 1:** Proximal composition (g 100 g^−1^ of flour) of native and extruded flours.

	RSF	CF	0 %eRSF	5 %eRSF	15 %eRSF
**Ash**	3.70 ± 0.06 ^d^	0.41 ± 0.06 ^a^	0.43 ± 0.06 ^a^	0.62 ± 0.06 ^b^	0.98 ± 0.06 ^c^
**Fat**	0.47 ± 0.06 ^a^	0.80 ± 0.06 ^b^	1.71 ± 0.06 ^d^	0.77 ± 0.06 ^b^	0.97 ± 0.06 ^c^
**Moisture**	5.44 ± 0.03 ^a^	13.22 ± 0.03 ^e^	9.01 ± 0.03 ^c^	8.79 ± 0.03 ^b^	9.17 ± 0.03 ^d^
**Protein**	6.88 ± 0.06 ^b^	6.56 ± 0.06 ^a^	6.81 ± 0.06 ^b^	6.81 ± 0.06 ^b^	6.88 ± 0.06 ^b^
**Carbohydrates**	83.51 ± 1.22 ^b^	79.01 ± 1.22 ^a^	82.04 ± 1.22 ^ab^	83.02 ± 1.22 ^b^	82.00 ± 1.22 ^ab^
**Fiber**	22.50 ± 0.11 ^e^	4.30 ± 0.11 ^c^	2.10 ± 0.11 ^a^	2.50 ± 0.11 ^b^	5.90 ± 0.11 ^d^
**Starch**	4.58 ± 0.07 ^a^	69.55 ± 1.25 ^c^	77.76 ± 3.00 ^d^	63.42 ± 1.78 ^b^	67.40 ± 0.64 ^c^

Abbreviations: RSF, Royal Summer flour; CF, corn flour; 0 %eRSF, 0% extruded Royal Summer flour; 5 %eRSF, 5% extruded Royal Summer flour; 15 %eRSF, 15% extruded Royal Summer flour. Different lowercase letters in the same row indicate significant differences in the mean values (one-way ANOVA, Duncan’s test, *p* ≤ 0.05) between the samples.

**Table 2:** Colorimetric parameters (CIE, L* a* b*) of native and extruded flours, with a particle size ≤ 500 µm.

	RSF	CF	0 %eRSF	5 %eRSF	15 %eRSF
L*	63.99 ± 0.95 ^a^	85.65 ± 3.48 ^d^	84.81 ± 1.69 ^d^	79.54 ± 1.47 ^c^	74.48 ± 0.79 ^b^
a*	10.46 ± 0.26 ^d^	4.09 ± 0.31 ^c^	3.84 ± 0.26 ^b^	3.37 ± 0.36 ^a^	3.92 ± 0.13 ^bc^
b*	14.61 ± 0.51 ^a^	22.41 ± 1.74 ^c^	24.86 ± 2.25 ^d^	20.92 ± 1.67 ^b^	19.54 ± 0.85 ^b^

Abbreviations: RSF, Royal Summer flour; CF, corn flour; 0 %eRSF, 0% extruded Royal Summer flour; 5 %eRSF, 5% extruded Royal Summer flour; 15 %eRSF, 15% extruded Royal Summer flour. Different lowercase letters in the same row indicate significant differences in the mean values (one-way ANOVA, Duncan’s test, *p* ≤ 0.05) between the samples.

**Table 3:** Phenolic profile assessed by HPLC–ESI–QTOF-MS.

	RSF1	RSF2	15%eRSF
**Cyanidin 3-o-galactoside/Cyanidin 3-o-glucoside/Petunidin 3-o-arabinoside**	64.9	36.8 ± 2.4	0.6 ± 0.1
**Caffeoylquinic acid**	n.d.	3.5 ± 0.1	n.d.
**(+)-Catechin**	1.3	1.2 ± 0.2	n.d.
**(−)-Epicatechin**	1.1	3.6 ± 0.02	n.d.
**Caffeoylquinic acid**	6.1	10.7 ± 0.2	0.1 ± 0.002
**Procyanidin dimer B**	n.d.	4.5 ± 0.4	n.d.
**Procyanidin dimer B**	n.d.	3.3 ± 0.1	n.d.
**3-Feruloylquinic acid**	n.d.	n.d.	0.8 ± 0.02
**Quercetin 3-o-rutinoside**	0.2	3.0 ± 0.2	0.3 ± 0.02

Abbreviations: RSF1, Royal Summer peel flour (first season); RSF2, Royal Summer peel flour (second season); 15%eRSF, 15% extruded Royal Summer flour; n.d., not detected.

**Table 4:** Functional properties of native and extruded flours.

	RSF	CF	0 %eRSF	5 %eRSF	15 %eRSF
**BD (g/mL)**	0.54 ± 0.00 ^a^	0.83 ± 0.01 ^c^	0.91 ± 0.01 ^e^	0.79 ± 0.00 ^b^	0.88 ± 0.02 ^d^
**OHC (mL/g)**	3.97 ± 0.02 ^a^	3.82 ± 1.03 ^a^	3.49 ± 0.01 ^a^	3.31 ± 0.59 ^a^	3.98 ± 0.02 ^a^
**WHC (mL/g)**	6.44 ± 0.01 ^d^	2.98 ± 0.00 ^a^	5.48 ± 0.02 ^c^	3.82 ± 0.29 ^b^	3.64 ± 0.25 ^b^
**SC (mL/g)**	0.00 ± 0.00 ^a^	0.00 ± 0.00 ^a^	0.00 ± 0.00 ^a^	0.00 ± 0.00 ^a^	2.94 ± 0.05 ^b^
**EC (%)**	20.99 ± 2.14 ^e^	7.41 ± 0.00 ^d^	1.85 ± 0.00 ^b^	3.70 ± 0.00 ^c^	0.00 ± 0.00 ^a^
**FC (%)**	50.00 ± 0.00 ^b^	50.00 ± 0.00 ^b^	0.00 ± 0.00 ^a^	0.00 ± 0.00 ^a^	0.00 ± 0.00 ^a^
**LGC (%)**	20.05 ± 0.01 ^c^	8.05 ± 0.05 ^a^	8.06 ± 0.02 ^a^	8.06 ± 0.08 ^a^	12.12 ± 0.01 ^b^

Abbreviations: RSF, Royal Summer flour; CF, corn flour; 0%eRSF, 0% extruded Royal Summer flour; 5%eRSF, 5% extruded Royal Summer flour; 15%eRSF, 15% extruded Royal Summer flour; AD, bulk density (g/mL); OHC, oil-holding capacity (mL/g); WHC, water-holding capacity (mL/g); SC, swelling capacity (mL/g); EC, emulsion capacity (%); FC, foaming capacity (%); LGC, least gelling concentration (%). Different lowercase letters in the same row indicate significant differences in the mean values (one-way ANOVA, Duncan’s test, *p* ≤ 0.05) between the samples.

**Table 5:** Hydrolysis index (HI) and predicted glycemic index (pGI) of native and extruded flours.

	HI (%)	pGI
**CF**	66.94 ± 1.75 ^a^	65.90 ± 1.51 ^a^
**0%eRSF**	90.30 ± 1.32 ^c^	86.04 ± 1.13 ^c^
**5%eRSF**	101.05 ± 0.40 ^d^	95.30 ± 0.35 ^d^
**15%eRSF**	81.08 ± 1.81 ^b^	78.09 ± 1.56 ^b^
**Bread**	100.00 ± 0.00 ^d^	94.40 ± 0.00 ^d^

Abbreviations: CF, corn flour; 0%eRSF, 0% extruded Royal Summer flour; 5%eRSF, 5% extruded Royal Summer flour; 15%eRSF, 15% extruded Royal Summer flour. Different lowercase letters indicate significant differences in the mean values (one-way ANOVA, Duncan’s test, *p* ≤ 0.05) between the samples.

## Data Availability

Data are provided within the manuscript.
